# Maximum parsimony xor haplotyping by sparse dictionary selection

**DOI:** 10.1186/1471-2164-14-645

**Published:** 2013-09-23

**Authors:** Abdulkadir Elmas, Guido H Jajamovich, Xiaodong Wang

**Affiliations:** 1 Department of Electrical Engineering, Columbia University, 500 W 120th St, New York, 10027 NY, USA; 2 Department of Radiology, Icahn School of Medicine at Mount Sinai, New York, 10029 NY, USA

## Abstract

**Background:**

Xor-genotype is a cost-effective alternative to the genotype sequence of an individual. Recent methods developed for haplotype inference have aimed at finding the solution based on xor-genotype data. Given the xor-genotypes of a group of unrelated individuals, it is possible to infer the haplotype pairs for each individual with the aid of a small number of regular genotypes.

**Results:**

We propose a framework of maximum parsimony inference of haplotypes based on the search of a sparse dictionary, and we present a greedy method that can effectively infer the haplotype pairs given a set of xor-genotypes augmented by a small number of regular genotypes. We test the performance of the proposed approach on synthetic data sets with different number of individuals and SNPs, and compare the performances with the state-of-the-art xor-haplotyping methods PPXH and XOR-HAPLOGEN.

**Conclusions:**

Experimental results show good inference qualities for the proposed method under all circumstances, especially on large data sets. Results on a real database, CFTR, also demonstrate significantly better performance. The proposed algorithm is also capable of finding accurate solutions with missing data and/or typing errors.

## Background

A human genome is a sequence of nucleotides that can differ from one individual to another (approximately 0.1% difference between any two individual) due to various reasons, such as insertions/deletions of fractions of the sequence on the genome or mostly the substitution/mutation of single nucleotides on commonly observed sites called *single nucleotide polymorphism* (SNP) [1]. In most SNPs only two different nucleotides are observed out of 4 nucleotides. The information of nucleotide variations extracted from these SNP sites (loci) is encoded as a sequence called “haplotype”. That is, for a particular SNP site a notation is used for one of the observed nucleotides (e.g., the most commonly observed nucleotide variant - *dominant/major allele*) and another notation is used for the other (e.g., the least observed nucleotide variant - *recessive/minor allele*). Because of its informative and heredity nature identifying the haplotypes of individuals has been an important subject in various medical and scientific studies, such as gene related disease discovery and drug design [2,3], population history research [4], etc. Nonetheless, current experimental techniques are not low-cost and efficient enough for directly sequencing haplotypes of an individual; thereby identifying them is mostly based on indirect approaches, e.g., using computational methods to infer haplotypes from an alternative cost-effective data called “genotype”.

The entire human genome consists of 23 distinct chromosomes each appearing in two copies (autosomes) except for the chromosome-23 (allosome) which consists of two copies of chromosome-X in females or one chromosome-X and one chromosome-Y in males. Each chromosome is a pair of two distinct sequences -haplotypes- inherited from the parents, i.e., one is from the maternal genome and the other is from the paternal genome. The genotype is sequenced by identifying the types of alleles -nucleotide variants- across the SNP locations (locus) in chromosomes. In a particular locus of a chromosome if both haplotypes have the same allele we call this site in the genotype *homozygous* and denote it with the type of alleles in both haplotypes as either common-type or wild-type; otherwise, if both haplotypes have different alleles –one common-type and one wild-type– we call this site *heterozygous*. When identifying haplotypes for a given genotype, the ambiguity occurs for the heterozygous sites since there is no information about which haplotype has the common-type allele and which haplotype has the wild-type allele. Clearly, genotypes are less informative than haplotypes, as they present an ambiguity on heterozygous sites due to possible permutations and computational methods can be employed to identify which allele come from which haplotype. Recently, more cost-effective alternative methods have been used for genotype sequencing [5], e.g., widely used *denaturing high-performance liquid chromatography* (DHPLC) [6]. By certain applications of such methods one can only determine whether an individual has homozygous or heterozygous allele in a given SNP site, but cannot distinguish the type of allele in homozygous sites. The sequenced data is thereby less informative than the regular genotypes as it only represents the differing sites (XOR operation) between the haplotypes. This less informative form of genotype is named *xor-genotype*. One can solve the haplotype inference problem based on the xor-genotypes, i.e., xor-haplotyping, with a reasonable extra computational effort.

Methods for solving the haplotype inference problem given the regular genotypes can be summarized in two categories: combinatorial methods that usually state an explicit objective function and propose methods for optimizing it, and statistical methods that relies on the statistical modeling of the problem. Various methods have been published for the haplotype inference problem [7-13], however the xor-haplotyping problem mostly remained under-investigated. Two particular methods are suitable for xor-haplotyping problems: parsimony haplotyping that is based on the maximum parsimony principle, and perfect phylogeny haplotyping that relies on a population genetics assumption called the *infinite sites/alleles model*[14], i.e., it assumes that allele sequences are long enough so that a particular allele will have a mutation only once in the phylogenetic tree. The perfect phylogeny (PP) model utilizes the infinite sites assumption by building a tree of individuals -haplotypes- where all individuals evolve, with no recurrent mutation, from one common ancestor. An approximate solution to xor-haplotyping problem in the case of PP model was introduced in [15] where the xor-haplotype inference was cast as a graph realization problem [16,17]. However, the proposed method (GREAL) in [15] is not well-suited for the xor-genotypes with large number of SNPs, i.e., usually limited by 30 SNPs [18], and is not extended to missing data cases.

On the other hand, it is known that in a population of individuals certain haplotypes are frequently found in certain genomic regions [19]. This fact leads to the *parsimony principle* that states that the genotypes of a population of individuals are generated by the least number of distinct haplotypes. Identifying such smallest set of haplotypes is called Pure (Maximum) Parsimony Problem, which is NP-hard [20]. An integer linear programing method was introduced in [21] that finds a pure parsimony solution to this problem, and in [22] a branch-and-bound method was used to solve pure parsimony problem. In [23] a method called XOR-HAPLOGEN was proposed for solving haplotype inference problem in the case of xor-genotype data. This method can find accurate solutions for xor-genotypes with large number of SNPs. Another parsimony method was introduced in [24] for xor-haplotype inference by representing it as a graph realization problem called pure parsimony xor haplotyping (PPXH).

In [25] a novel framework for (regular) haplotyping was proposed by interpreting the parsimony principle as a sparse representation of the genotypes. Two approaches are presented: maximizing a sparseness condition on the haplotype frequency vector determined by the inferred haplotypes, and casting the sparsity of this frequency vector as a sparse dictionary selection problem. The latter approach is based on an efficient greedy method SHSD where haplotypes explaining the given genotypes are determined according to a sparse selection from the set of compatible haplotypes. The method constructively determines the solution of each individual while selecting the haplotypes from this set, and it has the convergence guarantee.

For the xor-haplotyping problem, there is an increased ambiguity due to the XOR operation between haplotypes, i.e., the process of xor-genotyping that determines whether the type of alleles in both haplotypes differ in a particular site (heterozygous) or they are the same (homozygous). However this ambiguity can be resolved with the assistance of regular genotypes. Regular genotypes can either be used as post-processing inputs for eliminating set-equivalent solutions of a particular inference, or they can be used to refine inference while constructing the solution.

Tractability of the maximum parsimony haplotyping problem in the xor-genotype case is still open [24]. In this paper, we propose a modified version of SHSD —XHSD—, that can efficiently find a solution for maximum parsimony xor-haplotyping problem and resolve the ambiguity with the help of a small number of regular genotypes. For a given set of xor-genotypes the haplotype pairs for each individual are selected from the set of compatible haplotypes by a sparse dictionary selection method. The selection of dictionary columns from the set of compatible haplotypes and the sparse representation of xor-genotypes is formulated as a joint combinatorial optimization problem. The objective function of this problem maximizes a variance reduction metric over all individuals. Our algorithm is a low-complexity greedy method that terminates once the solution is fully determined. To resolve the ambiguity and to improve the inference accuracy, we employ a small number of regular genotypes as constraints for the set of compatible haplotypes to help resolve the type of homozygous alleles.

The remainder of the paper is organized as follows. In *Preliminaries*, we introduce the xor-haplotype inference problem. In *Methods*, we formulate the xor-haplotype inference as a sparse dictionary selection problem and present an efficient greedy method for solving this problem. We also discuss the use of regular genotypes to resolve ambiguity. In *Extensions* section we discuss how the algorithm deals with long sequences and data with missing sites. In *Results and discussion*, we present the experimental results on synthetic and real data sets under various conditions. Finally, the *Conclusions* section is given in the end.

### Preliminaries

In an SNP locus only 2 nucleotides are observed, and a single bit is sufficient for the representation of nucleotide variants such that 0 encodes the major allele and 1 encodes the minor allele. The haplotype of an individual can thereby be represented with a binary vector that shows the SNP variants across the individual’s chromosome. The genotype can then be thought of as a ternary vector where a 0 (2) indicates that the site is homozygous and both haplotypes have major 0/0 (minor 1/1) alleles, and 1 indicates that the site is heterozygous and the haplotypes have different alleles 0/1 or 1/0. Notice that when encoding homozygous and heterozygous sites we used a different notation from the literature in order to express a genotype vector as the sum of two haplotypes: a minor-homozygous SNP is encoded with 2 and a heterozygous SNP is encoded with 1, so that a 2 in the genotype is given by (the sum of) two minor alleles, and a 1 in the genotype is given by (the sum of) one major and one minor allele.

In general, given a length-*L* genotype vector, *k* ≤ *L* of the loci are heterozygous and thereby ambiguous, in each of the *k* sites one haplotype can take two values –0 or 1– and the other haplotype takes the complement value. Considering all *k* heterozygous sites, one haplotype can then be one of the 2^*k*^ binary sequences, and the other haplotype will be the complement (inverted values) of that sequence. Therefore, for solving a genotype with *k* heterozygous sites, the pair of haplotypes is drawn from a set of 2^*k*^ distinct binary vectors of length-*L*.

On the other hand, in xor-haplotyping problem the conflated data — *xor-genotype* — is less informative than the regular genotype with respect to the information loss about the type of allele in homozygous sites. The xor-genotype is itself a binary vector, where for a given site, 1 indicates heterozygous SNP where both haplotypes have different alleles for this given site. The xor-genotype can be represented by the XOR sum of two haplotypes, likewise, for a given site 0 indicates a homozygous SNP where the haplotypes have the same allele but without any distinction whether the type of the allele is major or minor. That is, the xor-genotype contains the information whether a particular SNP site has homozygous alleles, but the type of alleles for those homozygous sites is *not identified*. Every site of an xor-genotype is ambiguous, and each site of the corresponding haplotype can take two values. Therefore, a length-*L* xor-genotype can be explained by a pair of haplotypes that are drawn from a set of 2^*L*^ distinct binary vectors of length-*L*. Hence, because of the additional ambiguity on homozygous sites, the number of possible solutions for an xor-genotype is significantly (in fact, exponentially) larger than that of a regular genotype of the same size.

Besides the xor-haplotyping problem is NP-hard, there is also no unique solution to this problem. The nature of the XOR operation results in a phenomenon called *bit flip degree of freedom*[15], i.e., for a particular solution set ***H*** consisting of length-*L* haplotypes, one can produce equivalent solution sets by inverting a certain SNP *i* ≤ *L* (or a set of SNPs S⊆{1,…,L}) in all haplotypes of ***H***. Notice that inverting (complementing) an SNP across all haplotypes has no effect on the xor-genotypes they generated, because even the alleles explaining homozygous sites of xor-genotypes are not distinguished (hidden). More specifically, assume that $h_{i}^{1}(\ell )\in \{0,1\}$ and $h_{i}^{2}(\ell )\in \{0,1\}$ represent the haplotypes of *i*-th individual in the *ℓ*-th SNP and they generate that individual’s xor-genotype *x*_*i*_(*ℓ*) such that $x_{i}(\ell )=h_{i}^{1}(\ell )⊕h_{i}^{2}(\ell )$. Then the complemented SNPs of haplotypes also explain that SNP of the same xor-genotype, i.e., $x_{i}(\ell )=\bar{h_{i}^{1}(\ell )}⊕\bar{h_{i}^{2}(\ell )}$. It then follows that for a particular set ***H*** of length-*L* haplotypes that solves a given set of xor-genotypes, there are at most ∑i=1LLi=2L−1 equivalent sets $H_{i}^{\prime },\;i=1,\ldots 2^{L}-1$ to ***H*** where each equivalent set ***H****i*′ also solves that given set of xor-genotypes.

### Problem definition

For each SNP ℓ=1,…,L, the xor-genotype is given by the XOR-sum of two haplotypes such that,

(1) $$\begin{array}{l} x_{i}(\ell )=h_{i}^{1}(\ell )⊕h_{i}^{2}(\ell ),\;\ell =1,\ldots ,L, \end{array}$$

where *x*_*i*_(*ℓ*) ∈ {0, 1} is the xor-genotype of the *i*-th individual in SNP *ℓ*, and $h_{i}^{j}(\ell )\in \{0,1\}$ is the *j*-th haplotype of the *i*-th individual in SNP *ℓ*. Let $x_{i}=\;{\left[\;x_{i}(1)\ldots x_{i}(L)\right]}^{T}$ be the xor-genotype of the *i*-th individual, then (1) can be written as

(2) $$\begin{array}{l} x_{i}=h_{i}^{1}⊕h_{i}^{2} \end{array}$$

where $h_{i}^{j}=\;\;{\left[\;h_{i}^{j}(1)\ldots h_{i}^{j}(L)\right]}^{T}$ is the *j*-th (*j* = 1, 2) haplotype of the *i*-th individual consisting of *L* SNPs. In this representation, we say that the xor-genotype of the *i*-th individual ***x***_*i*_ is *phased* by the haplotype pair $\left\{h_{i}^{1},h_{i}^{2}\right\}$.

In regular haplotyping, a putative haplotype ***z*** ∈ {0, 1}^*L*^ is called *compatible* with a genotype ***g*** ∈ {0, 1, 2}^*L*^ if (***g*** − ***z***) ∈ {0, 1}^*L*^, and such a haplotype is a possible solution that can explain that genotype. That is, the haplotype pair {***z***,(***g*** − ***z***)} is one of the possible solutions to the genotype ***g***. Therefore, for every given genotype ***g***_*i*_ it is essential to determine a set of compatible haplotypes $H_{i}$ when searching for possible solutions. The union of the sets $H_{1},\ldots ,H_{N}$ for *N* individuals forms the matrix ***Z*** ∈ {0, 1}^*L* × *M*^ where *M* is the total number of distinct compatible haplotypes.

In xor-haplotyping, on the other hand, it is trivial to see that any haplotype ***z*** ∈ {0, 1}^*L*^ is *compatible* (consistent) with any xor-genotype ***x***, i.e., ***x*** = ***z*** ⊕ ***z***^′^ since there always exists a haplotype ***z***^′^ ∈ {0, 1}^*L*^ such that ***z***^′^ = ***x*** ⊕ ***z***. Therefore, the set of compatible haplotypes $H_{i}$ for a given length-*L* xor-genotype ***x***_*i*_ consists of all binary vectors of length-*L*, i.e., $H_{1}=H_{2}=\cdots =H_{N}={\left\{0,1\right\}}^{L\times 2^{L}}≜Z$.

Because of this compatibility between the xor-genotypes and candidate haplotypes an SNP site can always be explained by either of the two alleles, and thus unambiguous SNPs do not exist anymore. Notice that, in particular, an xor-genotype with all-homozygous SNPs is still ambiguous and requires to be solved up to bit flipping. However, we know that such an xor-genotype is always explained by a pair of identical haplotypes which correspond to the same column of ***Z***. On the other hand, if there is at least one heterozygous SNP in the xor-genotype then its phasing haplotypes are not identical and correspond to the different columns in ***Z***.

The xor-genotype of *i*-th individual is expressed as

(3) $$\begin{array}{l} x_{i}={\left(Z\;v_{i}\right)}_{2} \end{array}$$

where (.)_2_ represents the component-wise *modulo-2* operation, and ***v***_*i*_ ∈ {0, 1, 2}^*M*^, **1**^*T*^***v***_*i*_ = 2, is the sparse vector indicating the haplotype locations as the indices of the matrix ***Z*** of consistent haplotypes. Notice that the modulo-2 operation in (3) is equivalent to the XOR operation between the two haplotypes selected by ***v***_*i*_.

Given ***Z***, finding the indicator vector ***v***_*i*_ for an individual is equivalent to inferring its haplotype pair $\left\{h_{i}^{1},h_{i}^{2}\right\}$. The maximum parsimony principle suggests that a given set of xor-genotypes should be explained by the smallest number of distinct haplotypes. Therefore, given the set of xor-genotypes for *N* individuals $\left\{x_{i},\;i=1,\ldots ,N\right\}$, one needs to infer the haplotype pairs for each individual $\left\{h_{i}^{1},h_{i}^{2},\;i=1,\ldots ,N\right\}$, so that the union of all inferred haplotypes forms the smallest set as possible. In other words, the xor-haplotyping problem is to infer ***v***_*i*_, *i* = 1, …, *N*, given ***Z*** while selecting as few columns of ***Z*** as possible.

## Methods

### Xor Haplotyping by Sparse Dictionary Selection (XHSD)

If an (all-homozygous) xor-genotype is explained by only one haplotype, i.e., ***x***_*i*_ = ***h***^*s*^ ⊕ ***h***^*s*^, where the haplotype ***h***^*s*^ is the *s*-th column of ***Z***, then the indicator vector multiplies that haplotype by 2, i.e., ***v***(*s*) = 2 and ***v***(*j*) = 0 for $j=\{1\ldots 2^{L}\}\setminus \{s\}$. Otherwise, if the xor-genotype is explained by two different haplotypes $x_{i}=h_{i}^{m}⊕h_{i}^{n}$, *m* ≠ *n*, then they are indicated by the vector ***v*** such that ***v***(*m*) = ***v***(*n*) = 1 and ***v***(*j*) = 0 for $j=\{1\ldots 2^{L}\}\setminus \{m,n\}$. Hence, we can rewrite (3) in the following more compact form

(4) $$\begin{array}{l} x_{i}={\left(Z_{A_{i}}\;{\tilde{v}}_{i}\right)}_{2} \end{array}$$

where $A_{i}$ is a set of indices corresponding to the nonzero elements of ***v***_*i*_, $Z_{A_{i}}$ is the submatrix of ***Z*** consisting of the columns indexed by $A_{i}$, and $\tilde{v_{i}}$ is the non-zero elements of ***v***_*i*_.

For each observed xor-genotype ***x***_*i*_, the phasing haplotypes are located in columns of ***Z*** indexed by $A_{i}$. The union of these column indices, i.e., $D=\cup_{i}A_{i}$, forms the dictionary of the haplotypes that suffices to construct all given xor-genotypes. The maximum parsimony principle then dictates that the dictionary D should contain the least possible number of elements that can reconstruct all observed xor-genotypes. The set of haplotypes indicated by such a sparse dictionary D is given by $H=Z_{D}$, where $Z_{D}$ is the submatrix of ***Z*** consisting of the columns indexed by D. Then ***H*** is a solution set to the maximum parsimony haplotyping problem for the given set of xor-genotypes {$x_{i}\;,\;i=1,\ldots ,N$}.

To solve the xor-haplotyping problem, we choose the sparse dictionary D to minimize the average distance between the observed xor-genotypes and the closest approximations constructed by the haplotypes in $Z_{D}$. Since there is no prior information about the dictionary D and the indices $A_{i}$ for proper reconstruction of each xor-genotype, determining D and $A_{i}$ leads to a combinatorial problem. This joint-optimization problem can be efficiently solved by a greedy method that we will explain next.

For an observed xor-genotype the reconstruction accuracy can be interpreted as the Euclidean distance between the observation and its closest approximation, i.e.,

(5) $$\begin{array}{ll} L_{i}(A) & =\underset{{\tilde{v}}_{i}}{\text{min}}∥x_{i}-{\left(Z_{A}\;{\tilde{v}}_{i}\right)}_{2}{∥}^{2},\; \end{array}$$

where A represents the indices of haplotypes in ***Z*** used to approximate ***x***_*i*_. Notice that an exact solution will satisfy $L_{i}(A)=0$. For a given dictionary D, the indices $A_{i}$ for reconstructing each xor-genotype will be determined by restricting $A_{i}$ to be a subset of D such that

(6) $$\begin{array}{ll} A_{i} & =\arg\underset{A\subseteq D,|A|\leq 2}{\text{min}}L_{i}(A).\; \end{array}$$

The individual cost function in (5) is then translated into a fitness function associated with a given dictionary D, i.e.,

(7) $$\begin{array}{ll} F_{i}(D) & =∥x_{i}{∥}^{2}-\underset{A\subseteq D,|A|\leq 2}{\text{min}}L_{i}(A).\; \end{array}$$

Finally, the fitness value of D is averaged over all individuals to measure the overall reconstruction accuracy

$$\begin{array}{l} F(D)=\frac{1}{N}\overset{N}{\underset{i=1}{\sum }}F_{i}(D). \end{array}$$

For a given cardinality (sparsity) of *n*, the best dictionary is therefore given by

(8) $$\begin{array}{l} D_{n}^{*}=\arg\underset{|D|\leq n}{\text{max}}F(D), \end{array}$$

and the sparsest dictionary that is sufficient to reconstruct all observed xor-genotypes is determined by

(9) $$\begin{array}{l} D^{*}=\underset{n}{\text{min}}\left\{D_{n}^{*}:F(D_{n}^{*})=\frac{1}{N}\overset{N}{\underset{i=1}{\sum }}∥x_{i}{∥}^{2}\right\}. \end{array}$$

Notice that determining both D for a given *n* in (8) and A for a given D in (7) is a combinatorial problem. In [26], it is shown that such combinatorial problems can be approximately solved efficiently by a simple greedy method if the objective function satisfies a fundamental property called *submodularity*. In [25], it is shown that the dictionary selection problem for (regular) haplotype inference has a cost function that is approximately submodular, and when a greedy method is used to optimize this cost function it can efficiently find an approximate solution with a theoretical guarantee [27].

For xor-haplotype inference, on the other hand, the problem is fundamentally different. That is, the submodularity property may not hold for the cost function in (5) due to the XOR operation, and thereby the theoretical guarantee does not hold either for the greedy method. Nonetheless, we still use the similar greedy heuristic as SHSD in [25] in order to maximize the variance reduction metric in (5) over the set of observations.

In our algorithm Xor Haplotyping by Sparse Dictionary Selection (XHSD), we start with an empty dictionary set $D_{1}=\phi$. Then at each iteration *ℓ*, among the consistent haplotypes that are not already in dictionary $D_{\ell -1}$, i.e., in $Z\setminus D_{\ell -1}$, we iteratively add the haplotype that contributes to the dictionary $D_{\ell -1}$ with the maximal marginal gain. That is, at iteration *ℓ* the haplotype $h^{m}\in Z\setminus D_{\ell -1}$ is added to $D_{\ell -1}$ if it satisfies

(10) $$\begin{array}{l} m=\arg\underset{k\in \{1\ldots 2^{L}\}\setminus D_{\ell -1}}{\text{max}}F(D_{\ell -1}\cup \{k\}). \end{array}$$

To compute (10) requires solving (5) and (6) for each *k*. In (6) for each individual *i*, $A_{i}$ is found by computing the Euclidean distance (5) between ***x***_*i*_ and the possible reconstructions given by the pairwise xor-sum of all columns in $Z_{D}$, and picking the columns that minimize (6). Whenever indices $A_{i}$ yield zero in (5) we can explain that individual with the corresponding haplotypes in ***Z***, i.e., $x_{i}={\left(Z_{A_{i}}\;{\tilde{v}}_{i}\right)}_{2}$. The dictionary $D_{\ell }$ keeps growing until all xor-genotypes are explained, i.e., $F(D_{\ell })=\frac{1}{N}\overset{N}{\underset{i=1}{\sum }}F_{i}(D_{\ell })=\frac{1}{N}\overset{N}{\underset{i=1}{\sum }}∥x_{i}{∥}^{2}$.

Notice that in XHSD algorithm the number of compatible haplotypes |*Z*| exponentially increase in comparison to regular haplotyping problem with SHSD. However, –when available– we can reduce *Z* with respect to regular genotype information via utilizing them in the cost function (5). The necessary modifications are discussed in the next section *XHSD with regular genotypes*. Another fundamental difference in xor-haplotyping is that the xor-genotypes do not provide unambiguous genotype information which one can initialize the dictionary with corresponding haplotypes and improve the reconstruction accuracy. Nonetheless, with a bias weight, the modified cost function can exploit the available regular genotypes even when they are not unambiguous.

Summary of XHSD algorithm:

• Initialization. 

– $Z={\left\{0,1\right\}}^{L\times 2^{L}}$.

– *n* ← 1.

– $D_{n-1}^{*}=\phi$.

• Iterate until all xor-genotypes are explained, i.e., $F\left(D_{n}^{*}\right)=\frac{1}{N}\overset{N}{\underset{i=1}{\sum }}∥x_{i}{∥}^{2}$. 

– Perform the greedy search.

∗ For $\forall j\in \left\{1,\ldots ,2^{L}\right\}\setminus D_{n-1}^{*}$, compute $F\left(D_{n-1}^{*}\cup \{j\}\right)$.

∗ Let $j^{*}=\arg\;{\text{max}}_{j\in \{1\ldots 2^{L}\}\setminus \overset{*}{\underset{n-1}{D}}}F\left(\overset{*}{\underset{n-1}{D}}\cup \{j\}\right)$.

Set $D_{n}^{*}=D_{n-1}^{*}\cup \left\{j^{*}\right\}$.

∗ Check if any xor-genotype is explained by the addition of the new element $h^{j^{*}}$, i.e., if (5) is zero. If so, the inferred haplotype pair for the individual with such an xor-genotype is $\left[\;h^{j^{*}},\;x_{i}⊕h^{j^{*}}\right]$.

– *n* ← *n* + 1.

Given the xor-genotypes of a set of individuals, this algorithm finds the haplotypes of each individual based on the maximum parsimony principle.

As an example, consider the following demonstration. Let ***x***_1_,***x***_2_ and ***x***_3_ be the xor-genotypes of three individuals each corresponding to three SNPs, i.e.,

$$\begin{array}{l} x_{1}=\left[\begin{array}{c} 0\\ 0\\ 1 \end{array}\right],\;x_{2}=\left[\begin{array}{c} 0\\ 1\\ 0 \end{array}\right],\;x_{3}=\left[\begin{array}{c} 0\\ 1\\ 1 \end{array}\right].\; \end{array}$$

The set of compatible haplotypes for these individuals will consist of all length-3 binary vectors, i.e.,

$$\begin{array}{l} Z=\left[\begin{array}{c} 01010101\\ 00110011\\ 00001111 \end{array}\right]. \end{array}$$

After initializing ***Z***, and starting with empty dictionary $D_{0}$, the algorithm performs the greedy search by adding one haplotype from ***Z*** (with the maximal marginal gain) at a time. At iteration *n* = 1, (10) calculates *m* = arg max(0, 0, 0, 0, 0, 0, 0, 0) and *m* is randomly picked as 5 among the equal maximum values, then the corresponding haplotype ***Z***_5_ = [001]^*T*^ is added to the dictionary, i.e., $D_{1}=\;[\;5]$, and $Z_{D_{1}}=\left[\begin{array}{c} 0\\ 0\\ 1 \end{array}\right]$. Similarly, at *n* = 2, *m* = arg max(0.33, 0, 0.66, 0, 0, 0.33, 0) = 3 is calculated and the haplotype ***Z***_3_ = [010]^*T*^ is added to the dictionary, i.e., $D_{2}=\;[\;5,3]$, and $Z_{D_{2}}=\left[\begin{array}{c} 00\\ 01\\ 10 \end{array}\right]$. ***x***_3_ is explained by the addition of new haplotype, i.e., ***x***_3_ = [001]^*T*^ ⊕ [010]^*T*^, yet the other xor-genotypes are not explained. At *n* = 3, *m* = arg max(1.33, 0.66, 0.66, 0.66, 0.66, 1.33, 0.66) = 1 is calculated and the haplotype ***Z***_1_ = [000]^*T*^ is added to the dictionary, i.e., $D_{3}=\;[\;5,3,1]$, and $Z_{D_{3}}=\left[\begin{array}{c} 000\\ 010\\ 100 \end{array}\right]$. Other two xor-genotypes are explained by the new addition, i.e., ***x***_1_ = [001]^*T*^ ⊕ [000]^*T*^, ***x***_2_ = [010]^*T*^ ⊕ [000]^*T*^, and the algorithm converges at *n* = 3 via calculating $F(D_{3})=\frac{1}{3}\overset{3}{\underset{i=1}{\sum }}F_{i}(D_{3})=\frac{4}{3}=\frac{1}{3}\overset{3}{\underset{i=1}{\sum }}∥x_{i}{∥}^{2}$.

This simple example demonstrates how the proposed greedy approach can efficiently construct sparse solutions, where three xor-genotypes are explained by only three haplotypes within three iterations. Nonetheless, the solution set has the ambiguity of being one of the equivalent sets of the true solution due to the bit flip degree of freedom which should be resolved.

#### Resolving bit flip degree of freedom

In [15] it is shown that the xor perfect phylogeny problem can be solved up to bit flipping based on the characteristics of the given xor-genotypes. Let ***X*** ∈ {0, 1}^*L* × *N*^ be the xor-genotypes matrix of *N* individuals such that $X=\;[\;x_{1}\;x_{2}\ldots x_{N}]$. Denote *χ*_*i*_ as the set of heterozygous loci for the *i*-th individual, i.e., *χ*_*i*_ = {*ℓ*:*x*_*i*_(*ℓ*) = 1}, where *x*_*i*_(*ℓ*) is the *ℓ*-th SNP in ***x***_*i*_. If there exists a set of individuals I⊆{1,…,N} whose xor-genotypes have empty intersection, i.e., $\cap_{I}\chi_{I}=\phi$, then with the knowledge of regular genotypes $G_{I}\in {\left\{0,1,2\right\}}^{L\times |I|}$ of those individuals one can remove all bit flip degrees of freedom. The empty intersection indicates that an SNP will have homozygous allele in at least one of those individuals and therefore that SNP can be resolved by revealing the type of allele at the corresponding regular genotype. Following this, a post-processing method is suggested in [15] that can remove the bit flip degree of freedom across the loci where a set of xor-genotypes have empty intersection.

**Figure 1:**
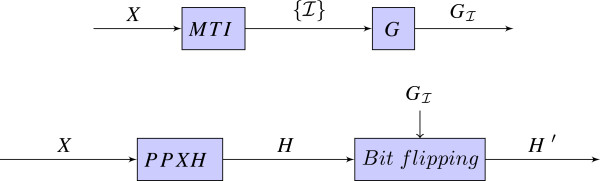
**Ambiguity resolution for PPXH method.** Informative regular genotypes $G_{I}$ are determined by the MTI algorithm, and they are used as control inputs for bit flipping on the initial inference result ***H***.

By bit flipping on a given solution ***H***, one attempts at choosing among the set-equivalent solutions $H_{i}^{\prime },\;i=\{1,\ldots ,2^{L}-1\}$ and this choice is decided by the given regular genotypes (Figure 1).

However, this post-processing method have certain limitations. Notice that, for large *L* the set-equivalent solutions are highly specific to the infererred ***H***, e.g., for a given set of xor-genotypes it is very likely that any two different inferences ***H***_1_ and ***H***_2_ —which are not set-equivalent— can have very different set-equivalent solutions. Bit flipping on different inferences likely leads to different results, and thereby the bit flipping accuracy largely depends on the initial inference ***H*** which is made by avoiding the prior knowledge on homozygous SNPs, i.e., regular genotypes. Besides, –when available– utilizing more regular genotypes in post-processing does not necessarily improve the bit flipping accuracy. Basically, to decide among the appropriate bit flippings for a particular locus requires the knowledge of that homozygous SNP from a regular genotype. Intuitively, to reveal a set of homozygous SNPs by employing the least number of regular genotypes, e.g., provided by the MTI method, will be necessary and sufficient for removing the bit flip degree of freedom across those SNPs. On the other hand, a larger number of regular genotypes will not be any more informative due to possible inconsistencies on the type of homozygous allele for an SNP site across the given regular genotypes.

Furthermore, notice that flipping the bits on some loci across all the haplotypes in ***H*** does not affect the parsimony of the solution. The final solution ***H***^′^ will have the same parsimony with ***H*** regardless of the set of loci that are flipped. From the maximum parsimony point of view, refining an xor-haplotyping solution via bit flipping method does not necessarily lead to global optimum unless the initial inference is a set-equivalent of the global optimal solution.

**Figure 2:**
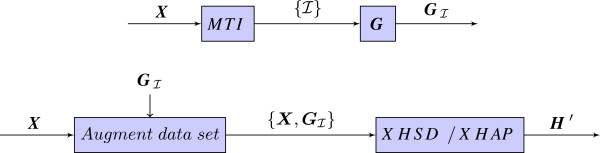
**Ambiguity resolution in XHSD or in XOR-HAPLOGEN (XHAP).** Informative regular genotypes $G_{I}$ are determined by the MTI algorithm, and they are used as inputs to augment the initial data set by replacing xor-genotypes of the individuals I⊆{1,…,N}.

Therefore, instead of using regular genotypes to post-process a solution, a more intuitive way could be to aim at resolving the bit-flip degree of freedom while constructing the solution. In particular, regular genotypes can be used as constraints when solving the homozygous sites of an xor-genotype. In this sense, given a set of individuals’ xor-genotypes we determine the individuals that have the most informative regular genotypes and pre-process the data set by replacing with the regular genotypes for those individuals. The MTI algorithm [15] is useful for finding the least number of such individuals that will be adequate to reveal the homozygous alleles for each of the *L* SNPs. In the proposed XHSD framework, we employ the MTI method to find which individuals should be replaced with regular genotypes and after replacing them the new data set is presented to the XHSD algorithm (Figure 2).

In most cases the xor-genotypes in ***X*** has empty intersection and for each run MTI outputs 2 or 3 individuals, i.e., |I|≤3; then $G_{I}$ has at most 3 regular genotypes. One can obtain a larger $G_{I}$ by performing multiple runs of MTI with ***X*** and collecting the distinct regular genotypes given by MTI.

Next we explain the necessary modifications to the XHSD algorithm for utilizing the regular genotypes.

#### XHSD with regular genotypes

The information provided by regular genotypes is used to reveal the type of allele in homozygous sites of an individual so that we can improve the reconstruction accuracy in (5), and build the dictionary D with more reliable haplotypes. That is, when a regular genotype ***g***_*i*_ is observed in the *i*-th individual we employ the variance reduction metric that is given for regular genotypes such that

(11) $$\begin{array}{ll} L_{i}(A) & =\underset{{\tilde{v}}_{i}}{\text{min}}∥g_{i}-Z_{A}\;{\tilde{v}}_{i}{∥}^{2}\;,\; \end{array}$$

where ***Z*** is the set of haplotypes that are compatible with the *i*-th individual’s genotype ***g***_*i*_, and A contains the indices of the haplotypes in ***Z*** that are used to approximate ***g***_*i*_. In this representation the approximation accuracy is potentially higher when compared to the xor-genotypes, since the homozygous SNPs in ***g***_*i*_ are unambiguous. The haplotypes that are used to approximate those SNPs will be more reliable candidates when building the dictionary D.

To exploit this fact, we can introduce a weight *b*_*i*_ in the cost function $L_{i}(A)$ so that the algorithm will give a higher priority on the variance reduction of those individuals that are given by regular genotypes, and the dictionary will more likely grow with the haplotypes that are compatible with the given regular genotypes. The biased variance reduction metric for each individual is then given by

(12) $$\begin{array}{ll} L_{i}(A) & =\left\{\begin{array}{c} b_{i}\;\;{\text{min}}_{{\tilde{v}}_{i}}∥g_{i}-Z_{A}\;{\tilde{v}}_{i}{∥}^{2},\;\text{given}\;g_{i}\\ {\text{min}}_{{\tilde{v}}_{i}}∥x_{i}-{\left(Z_{A}\;{\tilde{v}}_{i}\right)}_{2}{∥}^{2},\;\text{given}\;x_{i}\;. \end{array}\right)\; \end{array}$$

The weight parameter *b*_*i*_ could be set as proportional to the average rate of homozygous SNPs per genotype, assuming that the more homozygous sites the regular genotype contains the more informative it will be. We experimentally set *b*_*i*_=4 as it yielded good performance with both synthetic and real databases.

### Extensions

#### Long xor-genotypes

Note that the size of ***Z*** grows exponentially with the length-*L* due to the compatibility between haplotypes and xor-genotypes. That is, finding the solution of a length-*L* xor-genotype requires to perform the greedy search over ***Z*** that consists of 2^*L*^ haplotypes. To mitigate the computational complexity we employ the partition-ligation method [28] as in [25] where the block partitioning is based on identifying the recombination hot spots [29] existing between the haplotype blocks [30]. After partitioning the SNP sequences will be divided into blocks where within each block the haplotype diversity is as low as possible.

The haplotype diversity of a given block is measured by its Shannon entropy. The block partitioning by minimizing the total Shannon entropy proceeds as follows. Let $\left\{{\tilde{h}}_{1}^{\text{lm}}\ldots {\tilde{h}}_{{\tilde{K}}_{\text{lm}}}^{\text{lm}}\right\}$ be the ${\tilde{K}}_{\text{lm}}$ haplotypes that explains all the xor-genotypes $x_{i}^{\text{lm}},\;i=1,\ldots ,N$ in the block that starts at locus *l* and ends at locus *m*, i.e., 1 ≤ *l* ≤ *m* ≤ *L*, and let ${\tilde{f}}^{\text{lm}}=\left[{\tilde{f}}_{1}^{\text{lm}},\ldots ,{\tilde{f}}_{{\tilde{K}}_{\text{lm}}}^{\text{lm}}\right]$ be the haplotype frequency vector for this block. Each ${\tilde{f}}_{k}^{\text{lm}},\;k=1,\ldots {\tilde{K}}_{\text{lm}}$ is represented by the density of the nonzero values of the indicator vectors $\left\{v_{1}^{\text{lm}},\ldots ,v_{N}^{\text{lm}}\right\}$ for the given block $x_{i}^{\text{lm}}$, i.e.,

$$\begin{array}{lll} {\tilde{f}}^{\text{lm}} & ≜\frac{1}{2N}\overset{N}{\underset{n=1}{\sum }}v_{n}^{\text{lm}}\;.\; & \; \end{array}$$

The entropy of the haplotype block ${\tilde{h}}_{k}^{\text{lm}}$ is then given by

$$\begin{array}{lll} E(l,m) & =-\overset{{\tilde{K}}_{\text{lm}}}{\underset{k=1}{\sum }}{\tilde{f}}_{k}^{\text{lm}}\log{\tilde{f}}_{k}^{\text{lm}}\;,\; & \; \end{array}$$

and the total entropy of *Q* blocks, where each block $[\;l^{q}:m^{q}],\;q=1,\ldots Q$ has an upper bound of length *W*, i.e., *m*^*q*^ − *l*^*q*^ + 1 ≤ *W*, is given by

$$\begin{array}{lll} E & =\overset{Q}{\underset{q=1}{\sum }}E(l^{q},m^{q})\;.\; & \; \end{array}$$

To determine the initial and ending loci of each block $[\;l^{q}:m^{q}],\;q=1,\ldots Q$ that minimizes E we use the recursive method explained in [25], i.e., for each ending locus 1 ≤ *m* ≤ *L* we determine the block $\left[\;l_{m}^{*}:m\right]$, with $m-l_{m}^{*}+1\leq W$, that contributes with the lowest entropy and then backtrack the best initial points $l_{m}^{*}$ for each consecutive block by starting with the block $\left[\;l_{L}^{*},L\right]$.

#### Missing data

Genotyping errors often occur when the observed genotype of an individual differs from the original sequence for various reasons [31,32]. A particular type of genotyping error is the case when some loci are not observed/missed during sequencing or other application processes. Although methods dealing with some type of errors were proposed, often erroneous genotypes are produced with significant missing/error rates [33]. Therefore, it is of high importance for an xor-haplotyping technique to be adaptive for resolving such databases with missing sites. We next present a modification to XHSD in order to perform xor-haplotyping for the individuals exposed to missing data conditions.

Let ${\tilde{g}}_{i}$ be the incomplete genotype of the *i*-th individual where the loci with missing information in ***g***_*i*_ are removed. Similarly, let ${\tilde{x}}_{i}$ represent the xor-genotype of the *i*-th individual where the missing loci are removed. As the rate of missing loci increases the sequences become less informative. Following the suggestion in [25], we introduce another weight *w*(.) to give less weight to the less informative individuals when evaluating (12) in order to improve the reliability of haplotype inference, i.e.,

(13) $$\begin{array}{ll} L_{i}(A) & =\left\{\begin{array}{c} w({\tilde{g}}_{i})\;\;b_{i}\;\;{\text{min}}_{{\tilde{v}}_{i}}∥{\tilde{g}}_{i}-{\tilde{Z}}_{A}^{i}\;{\tilde{v}}_{i}{∥}^{2},\;\text{given}\;{\tilde{g}}_{i}\\ w({\tilde{x}}_{i})\;\;{\text{min}}_{{\tilde{v}}_{i}}∥{\tilde{x}}_{i}-{\left({\tilde{Z}}_{A}^{i}\;{\tilde{v}}_{i}\right)}_{2}{∥}^{2},\;\text{given}\;{\tilde{x}}_{i}\;. \end{array}\right)\; \end{array}$$

where ${\tilde{Z}}_{A}^{i}$ is the matrix ***Z*** with the rows corresponding to the missing loci of the *i*-th individual removed. The weight is selected as a nondecreasing function of the total information content in the sequence such that

(14) $$\begin{array}{l} w({\tilde{x}}_{i})=\dim{\left({\tilde{x}}_{i}\right)}^{2}\;, \end{array}$$

where $\dim({\tilde{x}}_{i})$ gives the dimension of ${\tilde{x}}_{i}$.

Different weight functions could be employed to exploit the distribution of missing sites. Since, in our experiments, the missing sites are uniformly distributed across the SNPs and individuals the function in (14) gave a good performance.

The proposed method does not account for the direct inference of the missing sites, i.e., imputing missing genotypes [34]. However, the missing values in each xor-genotype can be recovered from the solution by simply looking at the haplotype pairs which are specifically inferred for each individual. Since the proposed method has robust performance against missing data, as presented in the next section, the inferred solution will be sufficient to type missing genotype sites. An implementation of the proposed method –with aforementioned extensions– is provided in “Additional file 1”.

## Results and discussion

We tested the performance of several xor-haplotyping methods with a number of metrics. First we measured the *probability of error* (*P*_*e*_), i.e., the percentage of individuals whose inferred pair of haplotypes are different from the original pair. This measure is sensible for assessing the inference quality in regular haplotyping problem since the alleles corresponding to homozygous loci are known and only the heterozygous loci are ambiguous thereby performance depends on the inference accuracy on heterozygous loci. Nonetheless, in xor-haplotyping there are a large number of equivalent solutions to original one up to bit flipping and thereby it is very likely that a solution set differs from the original phasing on at least one SNP. In particular, for a given xor-genotype even if there is a single SNP difference (namely bit flip) between the pair of inferred haplotypes and the pair of haplotypes that originally gave rise to that xor-genotype, it is counted as mis-inference. A more sensible metric, therefore, would take into account the percentage of such SNPs where the inference differs from the true phasing. In that sense, the *switch error rate* (*swr*) [35] is a proper metric that counts the minimum amount of required switches for heterozygous loci to change to the correct alleles of the original haplotypes. It gives a sense of how closely the inference was made, i.e., as a ratio of total mis-inferred heterozygous loci $\text{mis}s_{i}^{\text{het}}$ in all individuals i={1…N} to the worst-case number of switches (half of the number of heterozygous loci in each individual *χ*_*i*_ / 2), i.e.,

$$\begin{array}{l} \text{swr}=\frac{\overset{N}{\underset{i=1}{\sum }}\text{mis}s_{i}^{\text{het}}}{\overset{N}{\underset{i=1}{\sum }}\frac{\chi_{i}}{2}}. \end{array}$$

Moreover, to assess the accuracy on homozygous sites, we employ *prediction error rate* (*err*_*p*_) [23] computed as the fraction of incorrectly predicted hidden-homozygous sites out of all hidden-homozygous sites, i.e.,

$$\begin{array}{l} \text{er}r_{p}=\frac{\overset{N}{\underset{i=1}{\sum }}\text{mis}s_{i}^{\text{hom}}}{\overset{N}{\underset{i=1}{\sum }}(L-\chi_{i})}. \end{array}$$

We performed xor-haplotyping on various data sets, with and without missing information on loci: synthetic data sets with different recombination rates simulated by a coalescence based program of [36], a database consisting of the SNPs in the CFTR gene that is associated with *cystic fibrosis* (CF) disorder [37], and another database (*ANRIL*) containing the SNPs that have relatively lower linkage disequilibrium (high polymorphism). We tested different xor-haplotyping methods that are based on different assumptions including the parsimony graph realization model PPXH [24], the parsimony genetic search model XOR-HAPLOGEN (XHAP) [23], the graph representation model GREAL [15], and an integer programming approach Poly-IP [38]. Among the four methods the last two were ineffective for practical reasons. GREAL failed at finding solutions for data sets with reasonably long sequences (SNPs >30), and Poly-IP method is often computationally inefficient when solving even a simple problem (e.g., it takes more than 24 hours to solve a set of 50 individuals with 30 SNPs).

### Synthetic data

Based on different recombination rates three different scenarios are considered in synthetic data sets: no recombination (*r* = 0), and recombination with rates *r*=4 and *r*=40, respectively. The recombination rate is the rate that the haplotypes of an individual exchange the sequence fragments due to several reasons such as crossing-over events. This fact is simulated by a model given in Hudson’s software [36]. For each scenario we generated 100 different data sets by random pairing of a set of simulated haplotypes of different lengths (5 ≤ *L* ≤ 46) for a given population size. This is repeated for different population sizes as well, *N* ∈ {10, 20, 30, 40, 50}.

**Figure 3:**
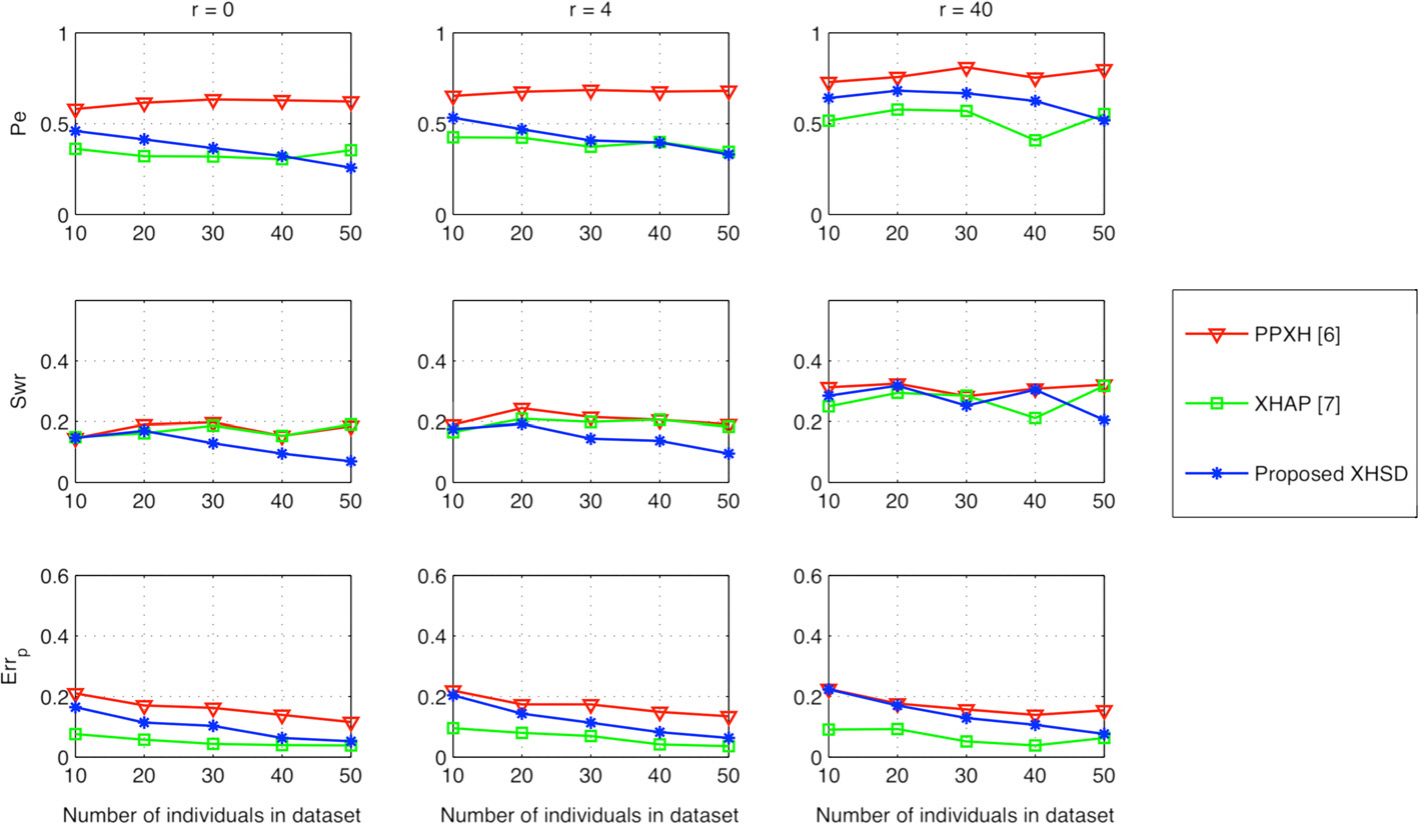
**Potential inference quality on short (*L *<14) synthetic data.**

In Figure 3, the performances of different methods on short data sets (*L*<14) are displayed which is based only on xor-genotypes. The quality of inference is exhaustively determined after removing all bit flip degrees of freedom by looking for the best equivalent set of a particular inference, i.e., performing an exhaustive search to find the best bit flipping that gives a result closest to the true phasing of xor-genotypes. Such evaluation shows the best inference performance of different methods without the help of regular genotypes. Compared to other methods, XHSD can potentially resolve a set of xor-genotypes with comparably low error rates. Moreover, XHSD achieves the lowest switch error rates, especially for large datasets, indicating a better accuracy (i.e., similarity with the true haplotypes) for the initial inference given only the xor-genotypes.

**Figure 4:**
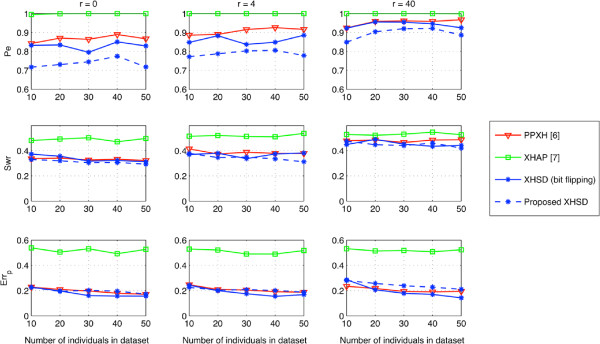
**Performance on long (5 *≤ L ≤ *46) synthetic data by bit flipping via 2 regular genotypes.**

To evaluate the inference quality when regular genotype data are available, we first determined only a limited number of regular genotypes by the MTI method, i.e., the smallest set of regular genotypes that have empty intersection on the heterozygous SNPs, then resolved the ambiguity by bit flipping on the initial inference according to these regular genotypes (Figure 4). This test evaluates how methods can deal with bit-flip degree of freedom under very limited regular genotype data that –in theory– suffice to resolve all SNPs. Given the long xor-genotype data sets (5 ≤ *L* ≤ 46), block partitioning is applied in XHSD by limiting the maximum block size to *W*=8 SNPs. From Figure 4, we can say that XHSD has the best potential to make an inference with high accuracy when the regular genotypes are introduced. We also applied the proposed XHSD framework represented in Figure 2 to the same dataset where 2 xor-genotypes are replaced with the regular genotypes. Note that the *Proposed XHSD* achieves a significant decrease in *P*_*e*_ rates despite the small augmentation of data by only 2 regular genotypes, compared to using them in the post-processing, i.e., *XHSD (bit flipping)*.

It is worthy of noting that the algorithms based on segmentation may deteriorate when processing long xor-genotype sequences, especially with increasing recombination rates where the detection of haplotype blocks is complicated [39]. We used block partitioning (segmentation) in XHSD to reduce complexity when processing long xor-genotype sequences. In Figure 4 the segmentation effect is noticeable particularly in very high recombination rates, i.e., *r* = 40. However, in general scenario, i.e., *r* ≤ 4, we can say that the segmentation effect is not significant for the proposed method’s performance, and it outperforms XOR-HAPLOGEN in most data sets containing typical recombination rates.

For more practical results we added regular genotypes in each method with different percentages of the population and allowed the methods to remove ambiguity by their own, except for PPXH. Since PPXH cannot make use of regular genotypes directly, we applied bit flipping using the MTI solver to remove ambiguity for this method. To regularly genotype a given percentage of the population, the regular genotypes are determined by running the MTI method several times until the number of distinct regular genotypes obtained achieves the given percentage of the total number of individuals.

**Figure 5:**
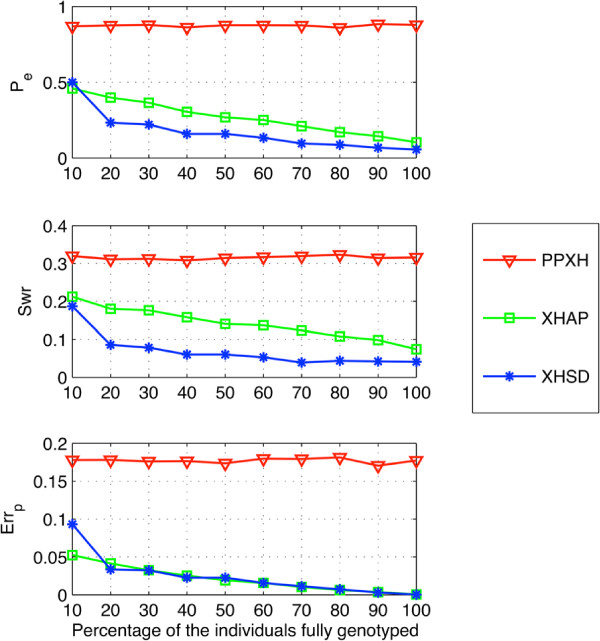
**Performance on long (5 *≤ L ≤ *46) synthetic data from 50 individuals by employing different numbers of regular genotypes.**

Figure 5 shows performances on the synthetic data of a large population of 50 individuals with zero recombination rate, where cases are considered from 10% (5 individuals) to 100% (50 individuals) of the population are given by regular genotypes. XHSD over-performs other methods in almost all cases. Particularly after 20% of the population is given by regular genotypes, XHSD can immediately utilize regular genotypes and significantly improve the accuracy on both homozygous (*err*_*p*_) and heterozygous sites (*swr*). We can conclude that the parsimony principle of XHSD method is well-suited for inferring the heterozygous sites, and for predicting the homozygous sites it usually suffices to have a small percentage of regular genotypes.

### Missing data

We investigated capability for dealing with missing data under different circumstances by various methods. Since the methods performed similarly under zero recombination rate we used the same data sets with no recombination to generate the database with missing entries. An SNP site of an individual is defined as “missing” with a probability of *P*_*miss*_ and the data sets for different percentages of missing SNPs are generated accordingly. PPXH method is excluded since it cannot handle missing data. In XHSD the block partitioning is applied as before with a maximum block size of *W* = 8 SNPs.

**Figure 6:**
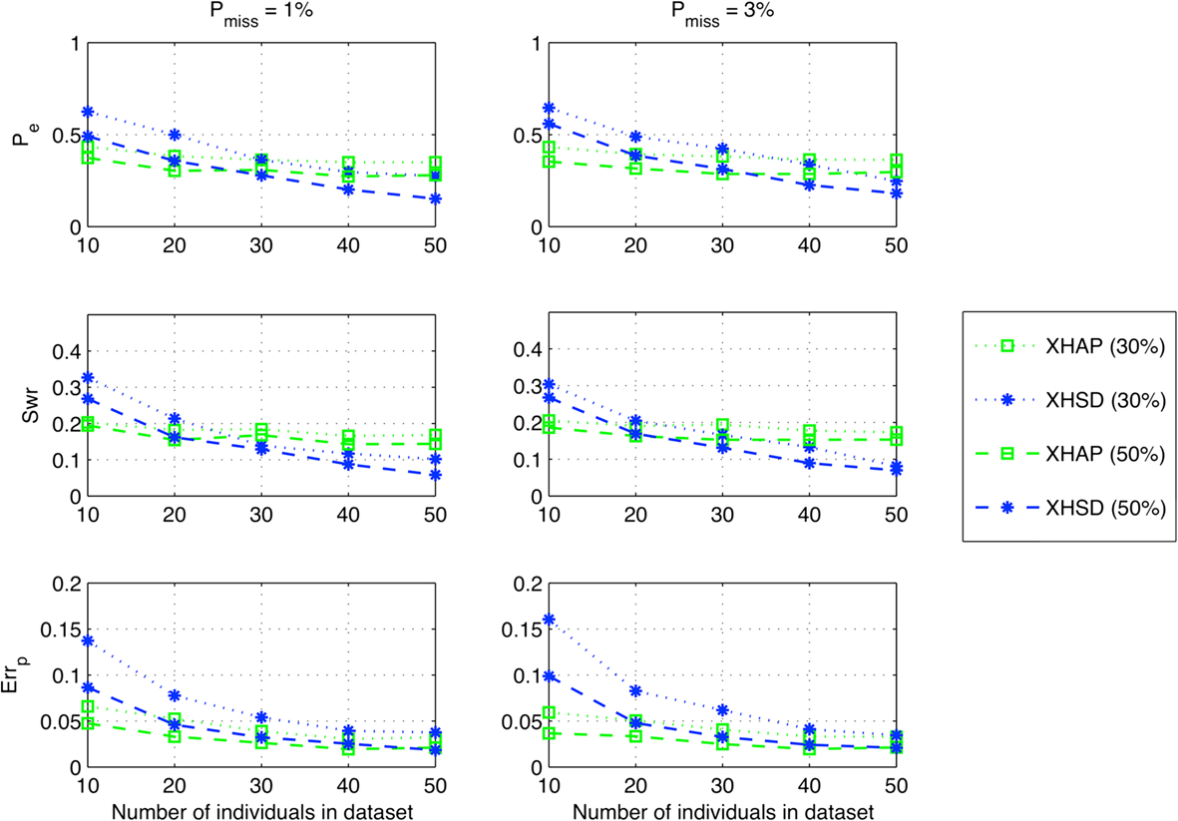
**Performance under low rates of missing data, long (5 *≤ L ≤ *46) synthetic data.**

**Figure 7:**
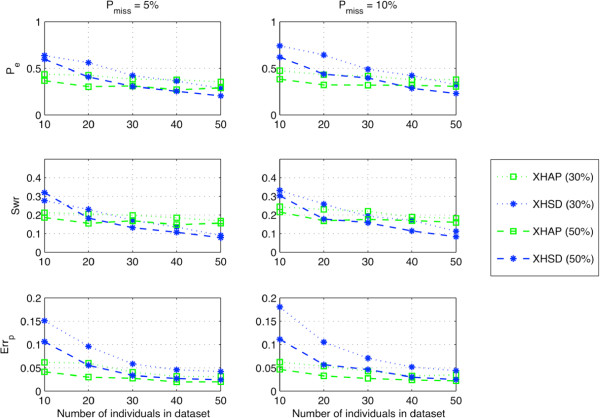
**Performance under high rates of missing data, long (5 *≤ L ≤ *46) synthetic data.**

Figures 6 and 7 show the performances in different scenarios of partial regular genotyping under different rates of missing data. As in the previous plots, each point represents the average value of the corresponding metric over 100 realizations–100 different sets of varying SNP sizes between 5 and 46. In most cases, XOR-HAPLOGEN and XHSD are insensitive to the increased number of missing sites. XOR-HAPLOGEN is more accurate for small group of individuals. Nonetheless, when more individuals are available in the database (*N*>30) XHSD displays a better performance in all circumstances.

**Figure 8:**
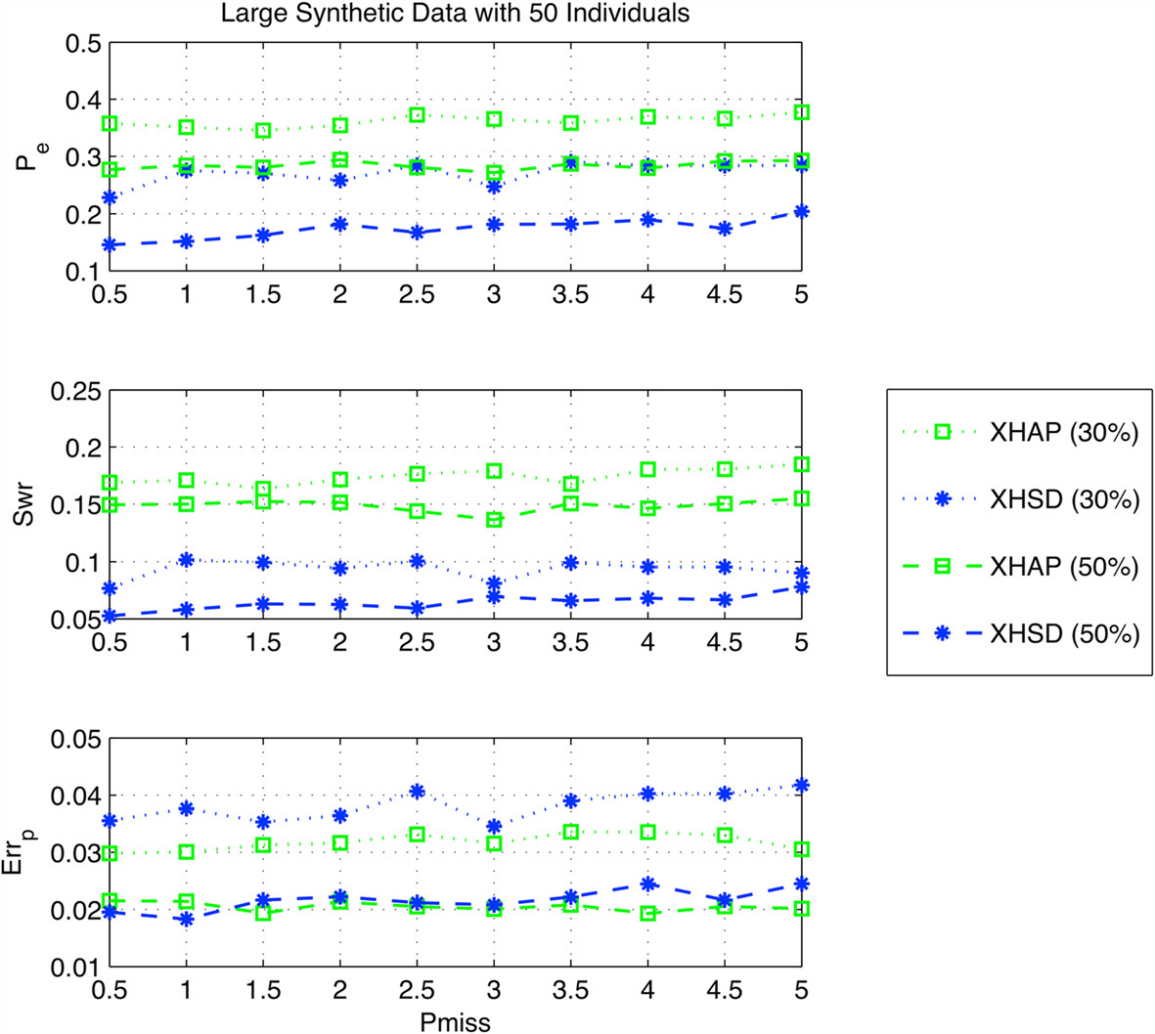
Performance under different percentages of missing data.

We examined the dependency of methods on percentage of the missing data rate for a population with large number of individuals. That is, we used the xor-genotypes from 50 individuals and replaced 30% and 50% of the population with regular genotypes, and performed xor-haplotype inference under different missing data rates ranging from 0.5*%* to 5%. As seen in Figure 8 both methods are robust against missing data. On the other hand, XHSD is less dependent on regular genotypes and it can achieve better error rates than XOR-HAPLOGEN by employing even less number of regular genotypes. XOR-HAPLOGEN needs approximately 20% more regular genotypes to reach the same *P*_*e*_ level with XHSD, e.g., regular genotyping by 30% in XHSD is comparable to that of 50% in XOR-HAPLOGEN.

### CFTR gene database

Cystic fibrosis (CF) is an autosomal recessive disorder caused by mutations in the gene that encodes the *cystic fibrosis transmembrane conductance regulator protein* (CFTR). In [37], various mutations on 23 polymorphic locations from the *chromosome 7* are detected as the disease loci for CF. We used this database corresponding to 29 distinct haplotypes to generate random xor-genotypes. By combining the haplotype pairs at random we generated the xor-genotypes for a given number of individuals *N*, and repeated the process for different population sizes, i.e., *N* ∈ {100, 200, 300, 400}. In this database, the data sets with small number of individuals present high haplotype diversities, i.e., many of the distinct haplotypes are only used once in the generation of individuals. Therefore, the larger data sets that have low haplotype diversities are expected to be solved with higher accuracy by biologically-oriented methods, such as XOR-HAPLOGEN which obtains its inference according to a multi-locus linkage disequilibrium (LD)-based block identification model.

**Figure 9:**
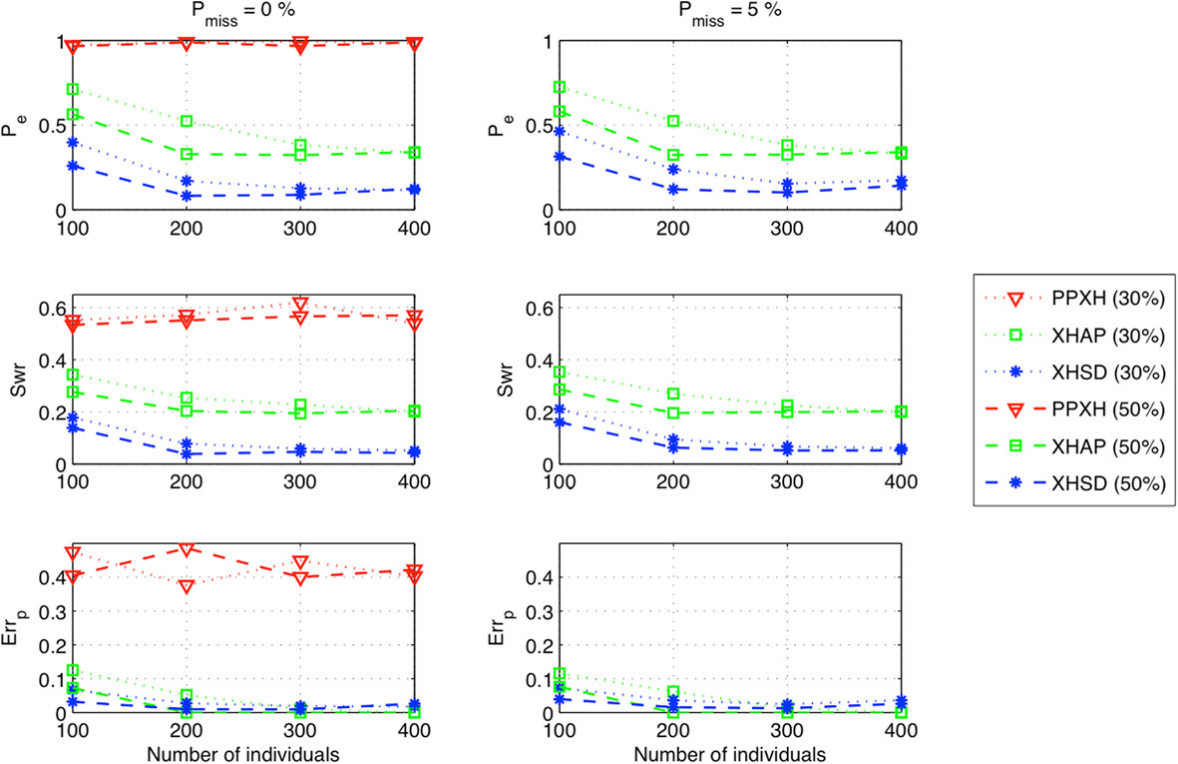
Performance on CFTR gene database with different population sizes with/without missing data.

We tested the performance of each method on this database with/without missing sites {0,5*%*}. PPXH method was excluded from the missing data analysis since it cannot deal with missing data. XHSD is applied with block partitioning and the maximum block length of *W*=8 SNPs as before. It is seen in Figure 9 that XHSD out-performs for various population sizes with significantly low error rates. As the xor-genotypes are taken from more individuals, the inference accuracy is immediately improved in XHSD and XOR-HAPLOGEN, whereas PPXH do not have this ability to benefit from the additional data.

**Figure 10:**
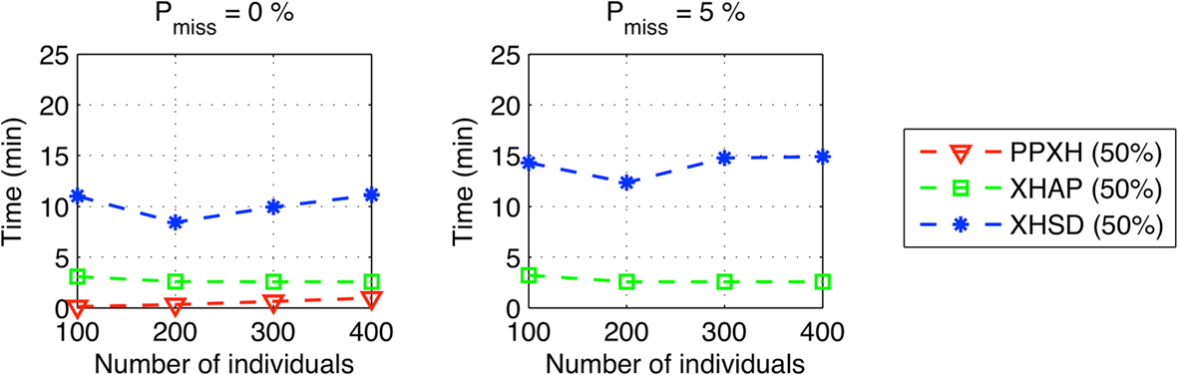
Running times on CFTR gene database with different population sizes with/without missing data.

Figure 10 shows the average running times of each method performing on this database. It is observed that XHSD has similar computational complexity as the size of data set grows, and it shows comparable running times with XOR-HAPLOGEN. Although PPXH performs significantly faster, it cannot mitigate the high error rates and is not able to provide accurate inferences.

### Typing errors

**Figure 11:**
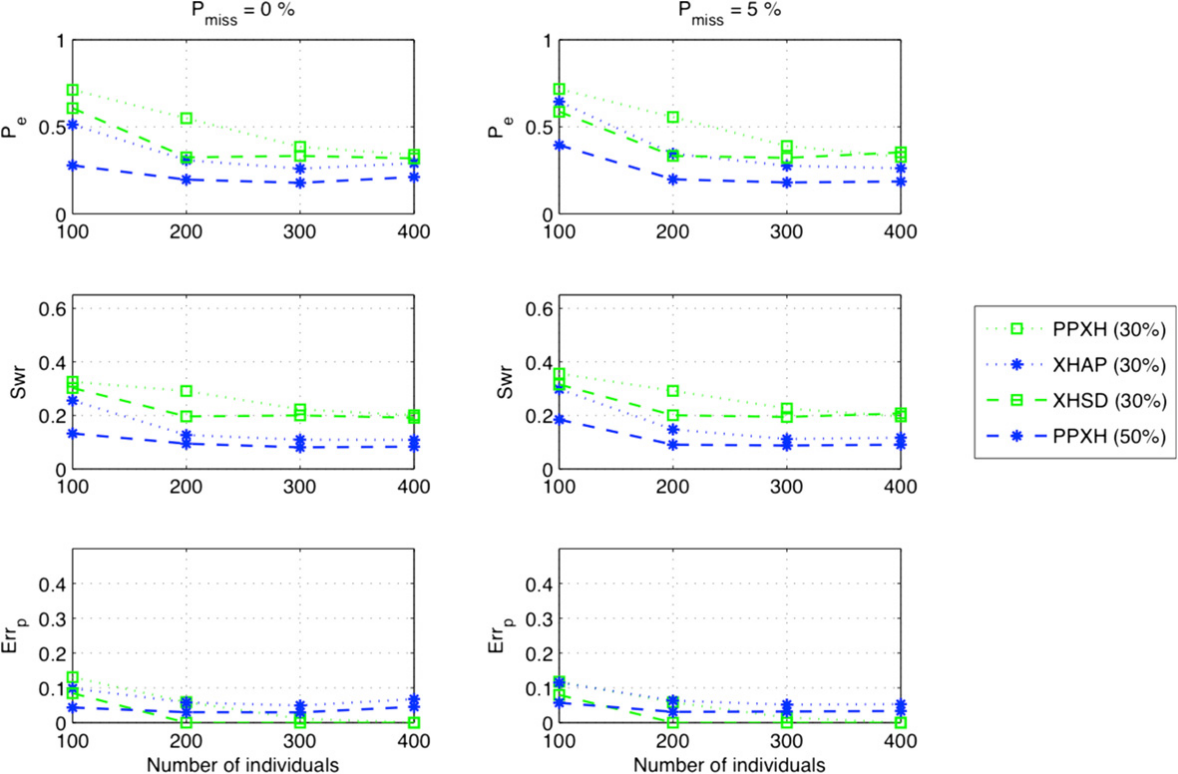
**Performance on CFTR gene database for different population sizes, with *P***_***err***_**=2%, with/without missing data.**

Combinatorial optimization techniques are known with their sensitivity to genotyping errors [40]. Thereby, we tested the effect of typing errors on the proposed algorithm using *CFTR gene database*. We defined a SNP site of an individual as erroneous with a probability of *P*_*err*_, and typed the site as either homozygous or heterozygous with equal probabilities. We then run the algorithms without providing the knowledge of erroneous site positions. We excluded PPXH method due to its low performance on the CFTR database. Figure 11 illustrates the algorithms’ performance on typing errors with *P*_*err*_ = 2*%*. It is seen that XOR-HAPLOGEN is a more robust method against typing errors because of its statical nature. Nonetheless, the proposed XHSD algorithm can deal with erroneous data containing ∼2% typing errors, with a small increase in the error rates compared to the results without typing errors.

### ANRIL database

**Figure 12:**
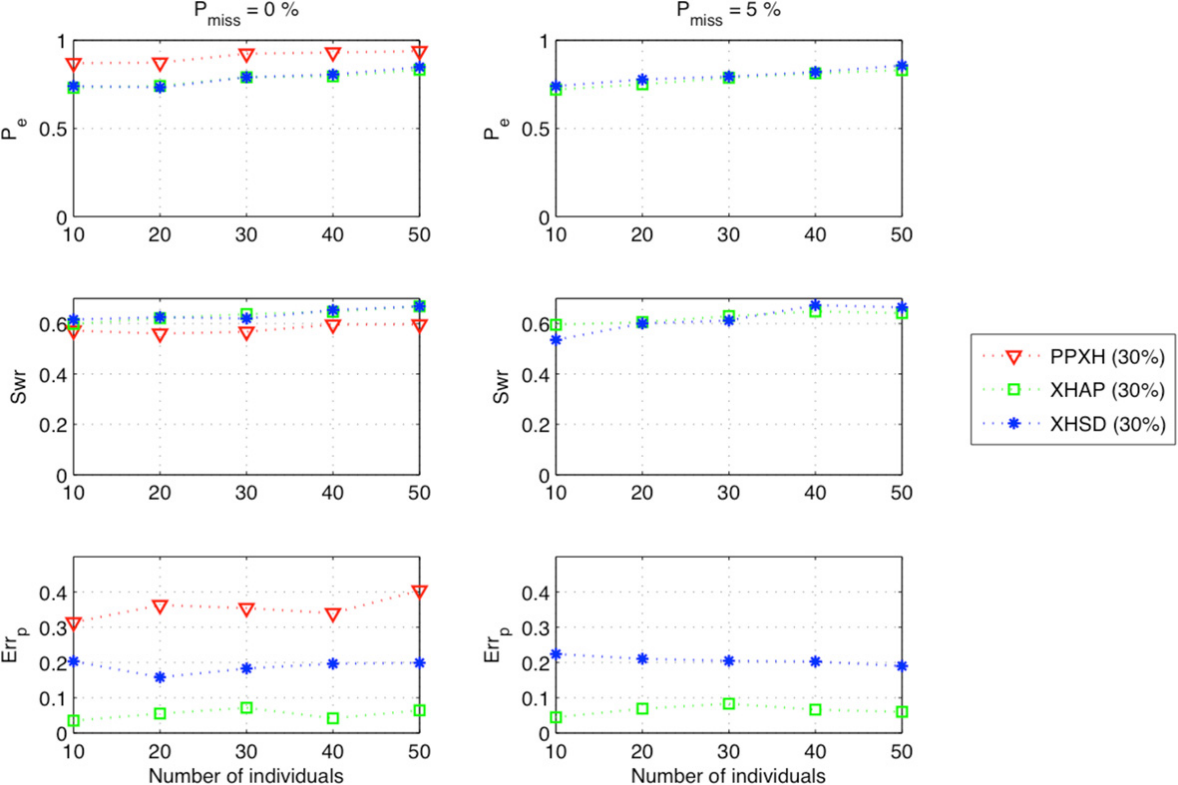
Performance on ANRIL gene database with different population sizes with/without missing data.

The performance of haplotyping methods can deteriorate on databases with decreasing linkage disequilibrium (LD) rates. A SNP database with low pairwise-LD scores are investigated in an association study given in [41] for their susceptibility to certain types of leukemia. This database includes 16 SNPs from the *chromosome 9p21* associated with several diseases and a SNP locus encoding for *anti-sense non-coding RNA in the INK4 locus (ANRIL)*[42]. We used the corresponding haplotype data from HAPMAP database (http://hapmap.ncbi.nlm.nih.gov/) collected from 90 European individuals. We generated the xor-genotypes for the individuals by using their haplotype pairs and tested the algorithms on this database. It is seen from the Figure 12 that the algorithms deteriorate when inferring the haplotypes with low-LD SNPs. XHSD shows very similar performance with XOR-HAPLOGEN, and both methods over-perform PPXH on this database.

Notice that the algorithms cannot mitigate the error rates with increasing number of individuals. This can be explained by the occurrence of very high haplotype diversity in corresponding low-LD SNP regions. The number of distinct haplotypes explaining the given number of individuals presumably remains at high diversity as the number of individuals grows, whereas the methods based on maximum parsimony principle fail to incorporate this fact. They are tend to find parsimonious (low-diversity) solutions in all population sizes, with a decreasing ratio (*ρ*) of “total number of distinct haplotypes explaining the given set of individuals” to “total number of given individuals” as the population size grows. It is worthy of noticing that, in XHSD results in Figure 12 (*P*_*miss*_ = 0), we observed that such ratio decreases as ***ρ*** = [1.3, 0.95, 0.83, 0.72, 0.66] in respect to the populations with 10,20,30,40,50 individuals; whereas the same ratio for the true phasing (ground truth data) is in fact much higher, i.e., ***ρ*** = [1.7, 1.48, 1.34, 1.27, 1.24], respectively, thereby causing the parsimony-based haplotyping methods to deteriorate on this database. On the other hand, in high-LD CFTR database, the same ratio for the true phasing is very low due to low haplotype diversity, i.e., ***ρ*** = [0.29, 0.14, 0.1, 0.07], in respect to the populations with 100,200,300,400 individuals, and the XHSD method is good at achieving very similar rates,i.e., ***ρ*** = [0.43, 0.15, 0.1, 0.07], respectively.

## Conclusions

In this paper, we have presented a new xor-haplotyping method XHSD based on the maximum parsimony principle that infers the haplotype pairs for each member of a group of unrelated individuals by observing their xor-genotypes. A dictionary selection method is utilized to find the smallest set of haplotypes selected from a candidate set that can explain the given set of xor-genotypes. The proposed approach requires regular genotypes from only a small percentage of individuals for the removal of ambiguity across all SNPs of the inferred haplotypes. The smallest subgroup of individuals having the most informative regular genotypes are efficiently determined by the minimum tree intersection algorithm. Although the inference accuracy was proportional to the percentage of the individuals given by regular genotypes, XHSD shows less dependency on regular genotypes compared to other methods. Experimental results have demonstrated that XHSD is a reliable method for xor-haplotyping under all circumstances including missing data and typing error cases. Low rates of missing values (≤ 10*%*) on the xor-genotypes has often insignificant contribution to the error rates, and the proposed method can deal with ∼ 2*%* typing errors. Particularly for large databases, XHSD produces the most accurate solution with significantly low error rates compared to other low-complexity xor-haplotyping methods. Experiments with CFTR gene database also proved that our approach can perform effectively on real data sets with/without missing sites. Another database with particularly lower LD rates indicates that the proposed algorithm can achieve the best performance with the state-of-the-art algorithms. We expect that XHSD can serve as a practical tool for xor-haplotyping on real-world large instances, as the large data collections become more available in the era of next-generation DNA sequencing.

## Competing interests

The authors declare that they have no competing interests.

## Authors’ contributions

XW and GJ conceived of the project. AE, GJ and XW participated in the design of the method. AE performed the computer experiments and contributed in the writing of the draft. All authors read and approved the final manuscript.

## Supplementary Material

Additional file 1 **Matlab implementation.** This file includes the Matlab code of the proposed algorithm, and an implementation with the example database, CFTR. Click here for file
