# Supplementary material for: Maximum parsimony xor haplotyping by sparse dictionary selection
Source: BMC Genomics. 2013 Sep 23;14:645. doi: 10.1186/1471-2164-14-645 (PMC3852077; doi:10.1186/1471-2164-14-645)
Supplement: Additional file 1 — Matlab implementation. This file includes the Matlab code of the proposed algorithm, and an implementation with the example database, CFTR. [file 1471-2164-14-645-S1.zip › XHSD method/ReadMe.pdf]

XHSD

This package contains the MATLAB implementation of the proposed xor-haplotyping algorithm.

Please see the script **textXHSD.m** to test XHSD algorithm on CFTR data with the desired parameters.

The main program is **sparsehaplotypeSPL.m**, which demands as input the genotype data. The genotypes should be located in the columns of the input matrix.

<u>INSTRUCTIONS for **saprsehaplotypeSPL.m**:</u>

Input Format: L x N matrix corresponding to N individuals with L SNPs are typed as follows.
0: homozygous-common 00
2: homozygous-mutant 11
1: heterozygous 01/10
4: homozygous-hidden (XOR-site) 00/11
6: missing SNP

Parameters:
W= maximum block size in PL method
interest= which individuals to infer haplotypes. Use [1, 2, .., N] as default.
Lmiss= L x N binary matrix where 1 means a missing site.
flagBlock= 1:PL method (default), 2:fixed-length partitioning, 0:no-partition

Output Format: L x N x 2 matrix.
Haplotypes are located in the corresponding columns in dimension 1 (L,N,1) and dimension 2 (L,N,2).
